# What Happens after Conservation and Management Donors Leave? A Before and After Study of Coral Reef Ecology and Stakeholder Perceptions of Management Benefits

**DOI:** 10.1371/journal.pone.0138769

**Published:** 2015-10-15

**Authors:** Timothy R. McClanahan, Nyawira A. Muthiga, Caroline Abunge, Albogast T. Kamukuru, Eliezer Mwakalapa, Hassan Kalombo

**Affiliations:** 1 Wildlife Conservation Society, Marine Program, Bronx, NY, United States of America; 2 Wildlife Conservation Society, Marine Program, Mombasa, Kenya; 3 University of Dar es Salaam, Department of Aquatic Sciences and Fisheries, Dar es Salaam, Tanzania; 4 Ministry of Livestock and Fisheries Development, Mtwara, Tanzania; 5 Regional Commissioner’s Office, Tanga, Tanzania; Instituto Español de Oceanografía, SPAIN

## Abstract

The coral reefs of Tanga, Tanzania were recognized as a national conservation priority in the early 1970s, but the lack of a management response led to damage by dynamite, beach seines, and high numbers of fishers until the mid 1990s. Subsequently, an Irish Aid funded IUCN Eastern Africa program operated from 1994 to mid 2007 to implement increased management aimed at reducing these impacts. The main effects of this management were to establish collaborative management areas, reduce dynamite and seine net fishing, and establish small community fisheries closures beginning in 1996. The ecology of the coral reefs was studied just prior to the initiation of this management in 1996, during, 2004, and a few years after the project ended in 2010. The perceptions of resource users towards management options were evaluated in 2010. The ecological studies indicated that the biomass of fish rose continuously during this period from 260 to 770 kg/ha but the small closures were no different from the non-closure areas. The benthic community studies indicate stability in the coral cover and community composition and an increase in coralline algae and topographic complexity over time. The lack of change in the coral community suggests resilience to various disturbances including fisheries management and the warm temperature anomaly of 1998. These results indicate that some aspects of the management program had been ecologically successful even after the donor program ended. Moreover, the increased compliance with seine net use and dynamite restrictions were the most likely factors causing this increase in fish biomass and not the closures. Resource users interviewed in 2010 were supportive of gear restrictions but there was considerable between-community disagreement over the value of specific restrictions. The social-ecological results suggest that increased compliance with gear restrictions is largely responsible for the improvements in reef ecology and is a high priority for future management programs.

## Introduction

The sustainable management of coastal fisheries resources is often challenged by the poverty of the resource users and weak capacity of the institutions that manage them [1]. Consequently, donor support has often been critical in catalyzing changes that are difficult to achieve where limited resources preclude implementing new ideas and actions [2]. Donor support is, therefore, frequently needed to extract resource users from poverty traps and promote more beneficial social-ecological relationships [3]. Nevertheless, the issues of suitability, adoption, compliance, beneficiaries, corruption, and the longer-term social-ecological and financial sustainability also challenge donor projects and generate considerable discussion without clear resolution [4]. Some of this consternation and poor resolution arises because donor projects are frequently motivated by social and environmental problems and international political concerns and possibly naiveté about existing values, social and ecological tradeoffs, informal institutional arrangements, and the capacity for change [5, 6]. Projects are therefore only seldom evaluated in a scientific way using a Before-After, Control-Impact (BACI) design [7, 8]. Donor projects are often site specific, time limited, and multi-faceted and this complicates simple single factor and well-replicated evaluations but they should, nevertheless, benefit from more rigorously applied impact evaluations.

There are many reasons and times when the donor-client relationship fails and the period when the donor is actively engaged is likely to be a prime time for reconsideration, change, reversion, abandonment, or a slow exit plan for the funded program. Donors may, at times, evaluate their projects during the various stages but when funds are spent and the project implementation phase is complete, the chances for full post-spending evaluation are considerably reduced. Further, evaluations seldom cover the full socio-ecological spectrum of variables and will most likely rely on perceptions of key stakeholders and consultants who place a stronger emphasis on easily collected social perceptions rather than more difficult-to-collect ecological metrics [9–11]. Yet, without an ecological assessment and an impact design that evaluates before, during, and after implementation, there is a considerable chance that evaluation will have limited ability to detect the effects of the implementation and gauge successes and failures [8, 12].

**Fig 1:**
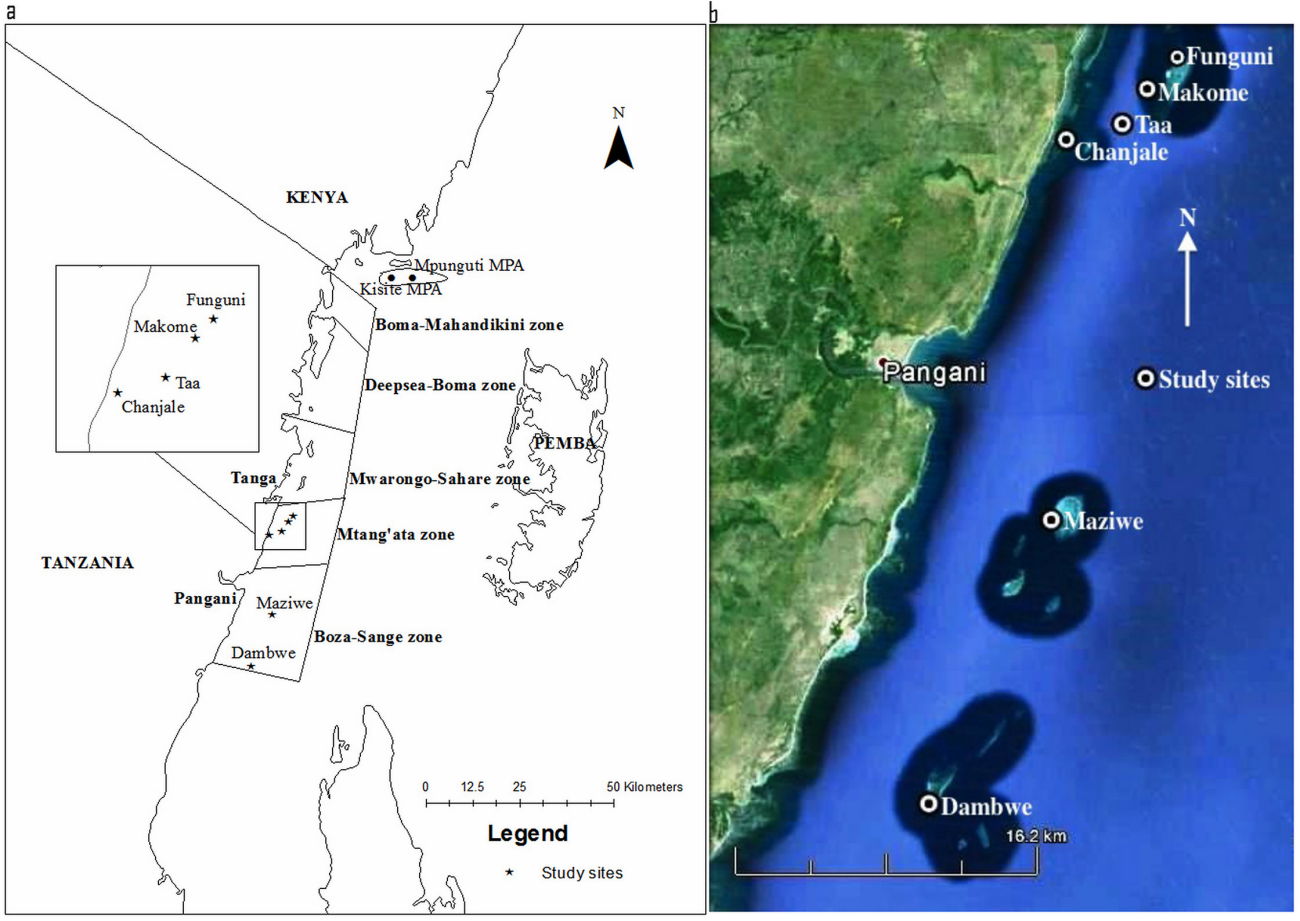
Map of the Tanga, Tanzania study location. (a) the Tanga coastline and collaborative management areas (CMA) of the coastal zone management project and (b) satellite image of reefs and study sites in the Mtangata and Boza-Sange CMA.

In this paper, we address this problem for a long-term and moderately-funded coastal zone project in Tanga, northern Tanzania where a variety of education and management efforts were made largely at the community level but also through integration of district and community level governance (Fig 1A). The management was primarily prompted by widespread dynamite fishing but also the desire to develop a system of management that would counter illicit gear use and lead to greater sustainability of a resource perceived to be in decline [13, 14]. Here, we evaluate changes in the benthic and fish communities in this region over a 15-year period that briefly predates the implementation aspect of the project that began in 1996 and evaluates the 14 year donor investment period three years after its termination. The working hypothesis based on various ecological studies and conceptual models of reef degradation would be that the increased management should lead to an increase in fish numbers and biomass of the most targeted fisheries species, their rates of predation and herbivory, a decline in sea urchin biomass, an increase in hard coral and calcifying algae, and a decline in non-calcifying algal abundance [15–17]. We also interviewed resource users after the project ended to determine the agreement between communities on the forms of management that they perceived to be most beneficial for sustainability.

## Materials and Methods

### Study sites and management history

Tanga has a coastline of about 180 km with ~100 distinct coral reefs of different sizes ranging from 0.5 km^2^ to 11 km^2^ (Fig 1A). The Tanga region has experienced a number of efforts at coastal and fisheries management, with first efforts being the colonial government’s Forest and Fisheries Acts followed by the post-colonial establishment of a system of district and village by-laws. The key Fisheries Act No. 6 of 1970 has been revised many times and was supplemented by the National Fisheries Sector Policy and Strategic Statement of 1997. In 1994 the Tanga Coastal Zone Conservation and Development Program (TCZCDP) was introduced with funding provided by the government of Ireland (Irish Aid). The program was implemented through the Tanzanian Government coastal districts with technical guidance from the IUCN Eastern African Regional Office (EARO). During the 12-year program, several initiatives were implemented but the principal efforts were focused on community involvement in fisheries management [18].

Through IUCN and the Tanzanian government’s facilitation and guidance, the communities suggested and implemented six collaborative management areas (CMAs), namely Boma-Mahandakini, Deepsea Boma, Mwarongo-Sahare, Mtang’ata, Boza-Sange and Mkwaja-Sanga, located from north to south (Fig 1A). The plans for these CMAs addressed several management issues and actions, including declining fish catch. Communities undertook to patrol their CMAs to reduce illicit gear use, specifically dynamite and seine nets, and closed some reefs to allow fish to recover. The study was undertaken at six reefs, Chanjale, Makome, Taa and Funguni in Mtangata CMA, and Dambwe, and Maziwe in Boza-Sange CMA (Table 1). Three of these reefs were closed during various times according to the areas CMA plans, Dambwe reef was closed in 1996, Makome in 2000 and although Maziwe was designated as a marine reserve in 1975, it was not closed until 2001 when communities finally supported the proposal to close the reef. In addition, in August 2009, an area of 552 km^2^ was declared, through community consultation, as the Tanga Coelacanths Marine Park (TACMP) that encompasses Chanjale, Taa and Tangoni reefs.

**Table 1:** The collaborative management areas (CMAs), year of establishment, the reefs within the CMAs and the year of closure of reefs within the CMA in the Tanga Region (a), and the physical characteristics of the study sites (b). The study sites and the villages adjacent to these sties are shown in bold.

a) Administrative characteristics of the study location						
**Year CMA established**	**Management Area**	**Villages adjacent to the CMAs**	**Reefs within the area**		**District**	**Year of reef closure**
1996	Boza—Sange	Kipumbwi, Boza, Sange, **Ushongo**, Pangani	Fungu Zinga, Masera kubwa, Masera ndogo, Mijimile ndogo, Mijimile kubwa, **Dambwe, Maziwe,** Makome Pangani, Dacha, Mwamba Mawe, Shuuni, Changuru, Chamgani,		Pangani	Dambwe 1996 Maziwe 2001
1996	Mtang’ata	**Kigombe**, **Mwarongo**, Tongoni, Geza, Maere.	**Makome, Funguni, Taa, Chanjale**, Kitanga, Upangu, Majivike, Shenguwe, Chamboni, Mgaa, Kange		Tanga/Muheza	Makome 2000
b) Physical characteristics of study reefs						
**Characteristic**	**Chanjale**	**Funguni**	**Makome**	**Taa**	**Dambwe**	**Maziwe**
Distance to shore, km	1.03	5.74	4.89	3.91	8.29	8.05
Depth, m	2	2	3	2	2	1.5
Exposure	Sheltered	Sheltered	Sheltered	Sheltered	Sheltered	Sheltered
Reef type	Patch	Patch	Patch	Patch	Patch	Patch
Rugosity	1.23 ± 0.07	1.31 ± 0.09	1.17 ± 0.08	1.24 ± 0.09	1.26 ± 0.07	1.28 ± 0.11

Tanga coral reefs are at the northern end of a high diversity center that extends from northern Mozambique to southern Kenya and is a region that may have some resilience to climatic impacts and potentially a refuge from climate change [19, 20]. Tanga reefs are isolated platform, banks, or rock island reefs that are located ~1–10 km offshore from the mainland (Fig 1B). The islands and banks are situated on the shallow continental shelf where the depth at the base of the reefs is less than 20 m deep. The studied reefs of Funguni, Makome, Taa, Chanjale, Maziwe and Dambwe are located adjacent to a rural area >20 km south of Tanga municipality that has a population of around 200 000 inhabitants. We studied the shallow water (<4 m at low tide where reefs have ~3.5 m tidal range) leeward sides of these rock island reefs that are dominated by living and dead coral colonized by various algae that is replaced by sand and seagrass at depths and shoreward (Table 1). These sites were chosen as being typical of this region and also being similar shallow back reef slopes with comparable coral reef lagoonal environmental characteristics. There are differences in the bottom complexity of the reefs and this can influence fish abundance and aggregations and their numbers and diversity, as well as the coral and algal community.

### Field methods

#### Ecological sampling methods

During three dry and warm periods (October to April), 1996 (April), 2004 (January), and 2010 (November), ecological sampling of fish, sea urchins, and benthic cover were undertaken in the same locations in four reefs, namely Chanjale, Funguni, Makome and Taa while Maziwe and Dambwe reefs were sampled in 2010 (November). Fish biomass in 20 families were counted in shallow water (<4 m at low tide) by a single pass of the belt transect where encountered individuals were categorized into family groups and 10 cm size class intervals with no individuals < 3 cm counted. The wet weight of the fish in each family group was estimated from length-weight relationships for these families [21].

Sea urchins were sampled in nine haphazardly placed 10m^2^ circular quadrats where all individuals were counted and recorded to the species level. Haphazard collections of ~20 individuals of each species were taken and their wet weights measured for converting population densities to wet weight biomass. Predation on sea urchins was measured by tethering the sea urchin *Echinometra mathaei* and recording mortality due to predators either fish or invertebrates, over a 24-hour period using the methods described in McClanahan [22]. This method produces a relative predation index where 0 is the lowest and 1 the highest rate, corresponding to no and all urchins eaten over the 24 hour period.

The benthic substrate was sampled using the standard 10 m line-intercept transect methodology [15]. Hard and soft coral, algal, fleshy, coralline and calcareous algae, seagrass, sponge and sand were measured along each transect and the percent cover was calculated for each substrate category per site. The topographic complexity of the bottom was estimated by the ratio of the bottom contour to straight-line distance using the same 10 m flexible transect line [23].

Herbivory was estimated by daily bite rates on a *Thalassia hemprichii* seagrass assay. Seagrass blades were cut to 9 cm and clipped to a weighted clothespin and attached to a thin nylon line at approximately 2 m intervals [24, 25] that was laid on the substrate at each site (depths ~3.5 m at low tide). Thirty clippings (10 per line) were deployed per site and left for 24 hours before retrieving and recording the remaining length (to the closest 0.5 cm) and the type of scar. Scar characteristics described by McClanahan *et al*., [25] allowed the observer to distinguish bites as originating from fish or sea urchins.

#### Socioeconomic field methods

Social surveys were completed in three fishing villages, Mwarongo, Kigombe, and Ushongo, in October 2010. These villages were adjacent to the fishing grounds that were sampled for the ecological variables. Villages were fishery dependent and composed of approximately 250 to 460 houses per village and a total of 76 fishers were interviewed. Interviews were predominantly conducted at the landing sites. In order to sample proportionally, the number of resource users at a site were determined from discussions with leaders and fishers during pre-survey meetings and the dominant fishing gear used by the fisher was used to classify the respondents.

Interviews were structured to determine the respondents’ views of sustainability and preferences for management using previously described methods [26]. Briefly, resource users were asked to list and rank the fishery that they exploit and the gear they use, the level of education, age, sex, place of origin and how long they had lived in the village, occupation and how long they had been fishing, job diversity, fortnightly expenditures and involvement in social /welfare groups and associations. Material style of life was also assessed based on the presence or absence of household possessions or structures [27].

Interviewees were asked about their level of agreement with various fisheries management options on a six point scale that included: agree completely, agree somewhat, neutral, disagree completely, disagree somewhat and don’t know. The six fisheries management options included closed areas (defined as those areas where fishing is restricted), protected areas (defined as multiple use areas that may include closures), seasonal closures, gear restriction, minimum fish size landed and species selection. The respondents were also asked the extent to which they perceived benefits of these management options to themselves, their community and the government by marking an X along a 10-cm ruler line that was scaled from low to high benefit. A measure of perceived disparity was also calculated as the difference between the perceived benefit for an individual and that for the government using the formula: Perceived mean disparity = (government benefit—community benefit) + (government benefit—self benefit)/2. This formula was derived after considering other logical options and a regional assessment of disparity [28]. Additional questions were asked on specific management options like appropriate area size for protected (under government jurisdiction) and closed areas (under community jurisdiction), period of closures for seasonal closures, and minimum size of fish that should be caught in cm.

#### Data analysis

Ecological data were first tested for normality using Shapiro-Wilk W Test. Benthic cover, fish, urchin and coral genera data did not meet the normality test requirement and therefore non-parametric Kruskal-Wallis tests were used to test for the difference between reef categories and time. Means, standard deviations of the means and the test of significances were calculated using JMP statistical software.

Interview data were analyzed by one-way ANOVA testing for difference in views between the occupations, fishers and managers, and villages. ANOVA was also used to test for differences between the village’s key descriptors such as respondent’s age, level of education, and fortnightly expenditures between the three villages. A Post-hoc Turkey test and ordinal logistic regression analysis were used to test whether the various management options had different levels of agreement among the three villages and for associations with the respondent’s socio-economic characteristics. Tests of autocorrelation between socio-economic variables indicated that many factors were auto-correlated and therefore a step-wise logistic regression was used and presented to distinguish the strongest factors.

#### Ethics statement

Permission to undertake this research was granted by the Board of trustees for marine parks and Reserves, Ministry of livestock development and Fisheries, United Republic of Tanzania. At the time the research was conducted, the Wildlife Conservation Society that was responsible for the research did not have an Institutional Review Board. Verbal consent was obtained from each participant before conducting the surveys and participants were informed about the purpose and the use of the data. Written consent was not obtained because of the low literacy rates of the participants.

## Results

### Ecological surveys

#### Finfish biomass

The biomass of fish varied with taxa (family), site, and time. For the reefs that were sampled in all three periods, the overall finfish biomass more than doubled from 260 kg/ha in 1996 to 772 kg/ha in 2010 (Table 2). The comparison between sites showed that damselfishes, surgeonfishes, wrasses and parrotfishes (in order of dominance) combined contributed more than 70% of the biomass in 1996. By 2010, these four families still contributed about the same percentage of the total biomass (69%) but the order of dominance changed with damselfish changing from most to least abundant and the wrasses contributing the largest proportion (35%) of the total biomass by the final sampling.

**Table 2:** The biomass of eleven common finfish families (mean ± SD, kg/ha) and the total finfish biomass at Chanjale, Funguni, Makome and Taa reefs that were sampled in 1996, 2004 and 2010 and the Kruskal-Wallis comparison of finfish families between sites and between sampling periods.

			Site					Site		Time	
Family	Chanjale	Funguni	Makome	Taa	1996	2004	2010	χ^2^	P	χ^2^	P
Surgeonfish	95.1 ± 84.2	72.8 ± 21.4	80.6 ± 75.5	140.0 ± 53.8	54.7 ± 18.9	82.5 ± 65.0	154.1 ± 57.1	4.4	0.217	10.9	0.004
Triggerfish	0	11.8 ± 18.4	34.2 ± 52.2	12.7± 13.2	0.2 ± 0.7	41.3 ± 39.9	2.5 ± 4.7	6.3	0.096	9.7	0.007
Butterflyfish	19.7 ± 10.0	10.0 ± 4.6	5.2 ± 5.3	3.6 ± 4.1	8.6 ± 4.0	11.1 ± 12.9	9.1 ± 8.2	11.7	0.009	0.04	0.981
Balloonfish	0	2.4 ± 5.8	0	2.4 ± 5.8	0	3.6 ± 6.6	0	2.1	0.554	4.2	0.124
Wrasses	67.8 ± 53.0	162.6 ± 126.1	52.2 ± 56.7	98.8 ± 91.3	39.0 ± 26.6	59.8 ± 28.1	187.3 ± 107.2	4.8	0.188	10.1	0.007
Snappers	3.6 ± 5.4	26.6 ± 26.2	4.0 ± 5.3	11.5± 13.2	4.2 ± 6.7	57.5 ± 55.0	43.4 ± 34.0	6.1	0.109	2.8	0.247
Goatfish	39.4 ± 32.0	69.9 ± 58.3	15.1 ± 21.0	36.1 ± 23.7	19.6 ± 12.8	7.7 ± 11.9	22.4 ± 23.5	4.6	0.201	6.2	0.046
Angelfish	16.6 ± 15.9	15.6 ± 10.0	5.6 ± 4.9	20.3 ± 19.7	5.2 ± 4.6	19.9 ± 16.8	18.4 ± 14.0	5.2	0.157	9.4	0.009
Damselfish	63.5 ± 20.3	78.0 ± 41.8	59.6 ± 41.1	60.9 ± 26.7	68.4 ± 13.9	54.6 ± 35.8	73.5 ± 41.8	0.7	0.873	0.5	0.797
Parrotfish	28.3 ± 40.3	165.1 ± 229.3	42.0 ± 26.8	46.3 ± 27.7	25.8 ± 21.4	68.3 ± 61.2	117.1 ± 203.0	4.1	0.250	3.9	0.143
Rabbitfish	6.4 ± 5.9	46.2 ± 68.3	0.3 ± 0.5	35.1 ± 81.4	2.5 ± 2.6	5.6 ± 7.2	57.9 ± 84.3	8.6	0.036	0.2	0.900
***Grand total***	***362*.*5* ± 201.8**	***741*.*8* ± 523.6**	***359*.*4* ± 235.4**	***512*.*3* ± 176.3**	***260*.*3* ± 72.4**	***450*.*0* ± 198.0**	***771*.*6* ± 415.8**				

The temporal changes in biomass of different finfish families were variable and exhibited highly significant increases for the surgeonfishes, triggerfishes, wrasses, goatfishes and angelfishes, and somewhat less significant increases in the ballonfishes and parrotfishes (Table 2). The magnitude of increase differed depending on the family group and the largest overall increases occurred in the rabbitfish family that had very low biomass in 1996 and which increased by 2200%, followed by the triggerfishes (1150%), wrasses (380%) and parrotfishes (350%). While most of the fish families showed a steady increase over time, the triggerfishes exhibited a large pulse in 2004 (4 orders of magnitude increase from 1996) attributed to the species *Sufflamen fraenatus*.

There was also variability and changes in biomass between reefs overtime. Funguni reef had on average the highest biomass and the greatest increase in biomass between 1996 and 2010 while Makome reef had the lowest overall biomass and Taa reef the lowest increase overtime. Comparison of differences in biomass of the different family groups between reefs showed significant differences in the butterflyfishes and rabbitfishes and less significant differences for the surgeonfishes, wrasses, snappers, angelfishes and parrotfishes (Table 2).

The finfish biomass at Dambwe and Maziwe reefs that were only sampled in 2010 (Table 3), showed similar overall biomass of finfish at the two reefs (~595 kg/ha and 696 kg/ha respectively) that were on average approximately 16 and 36% (respectively) higher than at Makome reef, approximately equal to Chanjale and Taa reefs but lower than at Funguni (~114 and 83% respectively) reef, which had the highest overall biomass of all the reefs in 2010 (Table 3). The dominance of the different family groups also differed, while parrotfishes, surgeonfishes, damselfishes and wrasses dominated at Maziwe reef, which was similar to other studied reefs in 2010, rabbitfishes, wrasses and damselfishes dominated at Dambwe reef. This reef also had the highest biomass of rabbitfishes and wrasses of all the studied reefs, while Maziwe had the highest biomass of parrotfishes and damselfishes (in order of dominance) of all the studied reefs in 2010 (Table 3). A comparison of the different family groups in 2010 showed marginally significant differences between reefs for the triggerfishes, butterflyfishes and surgeonfishes.

**Table 3:** The biomass of eleven common finfish families (mean ± SD, kg/ha) and the total finfish biomass at all reefs sampled in 2010 and the Kruskal-Wallis comparison of finfish families between sites.

Family	Chanjale	Funguni	Makome	Taa	Dambwe	Maziwe	χ^2^	P value
Surgeonfish	196.5 ± 58.1	93.5 ± 9.8	162.1 ± 87.9	164.4 ± 19.1	49.6 ± 15.3	154.3 ± 33.3	10.2	0.069
Triggerfish	0	0	1.0 ± 1.5	8.8 ± 6.5	2.1 ± 2.1	10.6 ± 7.3	10.9	0.053
Butterflyfish	19.5 ± 1.3	13.1 ± 0.2	2.0 ± 2.8	1.9 ± 2.7	13.7 ± 7.8	25.0 ± 9.9	10.6	0.060
Balloonfish	0	0	0	0	0	2.7 ± 5.4	2.6	0.739
Wrasses	132.6 ± 11.1	319.7 ± 51.3	84.9 ± 111.0	212.1 ± 46.9	137.9 ± 70.6	100.8 ± 9.7	9.1	0.106
Snappers	1.2 ± 0.1	50.6 ± 31.2	10.6 ± 2.9	27.2 ± 8.6	10.7 ± 9.3	19.4 ± 18.5	8.0	0.155
Goatfish	77.9 ± 5.5	56.4 ± 39.6	23.8 ± 33.7	15.39 ± 18.2	2.6 ± 0.5	3.1 ± 3.7	8.3	0.143
Angelfish	31.8 ± 21.3	22.0 ±10.3	4.1 ± 3.0	15.8 ± 1.4	27.2 ± 38.1	24.7 ± 6.7	5.7	0.333
Damselfish	64.5 ± 3.1	91.2 ± 74.2	57.2 ± 71.8	81.0 ± 12.6	93.5 ± 33.3	108.2 ± 58.5	2.5	0.778
Parrotfish	60.7 ± 68.7	305.9 ± 432.7	60.7 ± 28.2	41.2 ± 11.8	42.2 ± 55.6	170.5 ± 91.8	3.8	0.580
Rabbitfish	6.9 ± 9.7	124.0 ± 70.5	0	100.6 ± 142.3	179.9 ± 167.7	0	7.3	0.201
***Grand total***	***606*.*2 ± 53*.*4***	***1273*.*5 ± 620*.*7***	***510*.*9 ± 337*.*5***	***695*.*9 ± 47*.*5***	***594*.*5 ± 242*.*6***	***695*.*9 ± 80*.*2***		

Although no systematic information on compliance of the closures was available, a general comparison of the differences between the biomass of finfish in the closed and the open access reefs in 2010 showed variable results with no clear correlation between length of closure and finfish biomass. Of the open access reefs, Makome had the lowest biomass while Funguni had the highest biomass in 2010 (Table 3). Moreover, the oldest closure Dambwe (14-year closure) had a finfish biomass that was only slightly more than half of that of Funguni—an open access reef. Finfish biomass at Makome (10-year closure) was lower than at Maziwe (9-year closure) while the latter reef had finfish biomass that equaled that at the open access reef Taa and was higher than at Chanjale reef.

#### Finfish sizes

Comparison between the biomass of different size classes of fish showed significant spatial and temporal variation among sites (Table 4). On average across the sites that were studied in all three periods, the largest changes over time were recorded in the larger size classes where there was a 74% increase in the 10–20cm, 644% increase in the 20–30cm, and an 7331% increase in the 30–40cm size classes. These increases were statistically significant for the 20–30cm and 30–40cm size classes. There was also a significant decrease in the smallest size classes (3–10cm) between 1996 and 2010 (p = 0.039). The comparison between sites showed a significant difference in the 10–20cm and >40cm size classes.

**Table 4:** The biomass finfish (mean ± SD, kg/ha) of different size classes at Chanjale, Funguni, Makome and Taa, that were sampled in 1996, 2004 and 2010 and results of Kruskal-Wallis tests of differences between reefs and years.

		Sites				Time			Site		Time
Size class	Chanjale	Funguni	Makome	Taa	1996	2004	2010	χ^2^	P	χ^2^	P
3–10 cm	62.8 ± 36.4	55.1 ± 19.2	68.3 ± 43.1	61.8 ± 31.4	84.2 ± 26.0	55.2 ± 26.2	46.5 ± 32.6	1.3	0.729	6.5	**0.039**
10–20 cm	109.4 ± 32.5	250.1 ± 138.2	99.8 ± 58.2	172.2 ± 71.6	128.2 ± 55.7	122.8 ± 38.6	222.7 ±145.1	10.2	**0.0169**	3.0	0.225
20–30 cm	141.3 ± 143.2	278.5 ± 270.1	101.6 ± 109.2	184.6 ±120.7	46.4 ± 37.1	138.0 ± 97.6	345.2 ±189.8	2.7	0.444	15.7	**0.0004**
30–40 cm	31.7 ± 56.7	155.5 ± 184.6	81.7 ± 106.6	67.5 ± 71.1	1.6 ± 4.5	131.7 ±102.8	118.9 ± 151.6	1.9	0.592	10.3	**0.006**
>40 cm	5.7 ± 11.0	12.3 ± 19.5	0	26.6 ± 34.5	0	9.7 ± 13.7	23.7 ± 32.1	4.0	**0.026**	5.1	0.075
***Totals***	***350*.*9 ± 189*.*4***	***751*.*5 ± 519*.*7***	***351*.*3 ± 247*.*4***	***512*.*7 ± 176*.*5***	***260*.*4 ± 72*.*5***	***457*.*4 ± 202*.*9***	***757*.*0 ± 432*.*8***				

**Fig 2:**
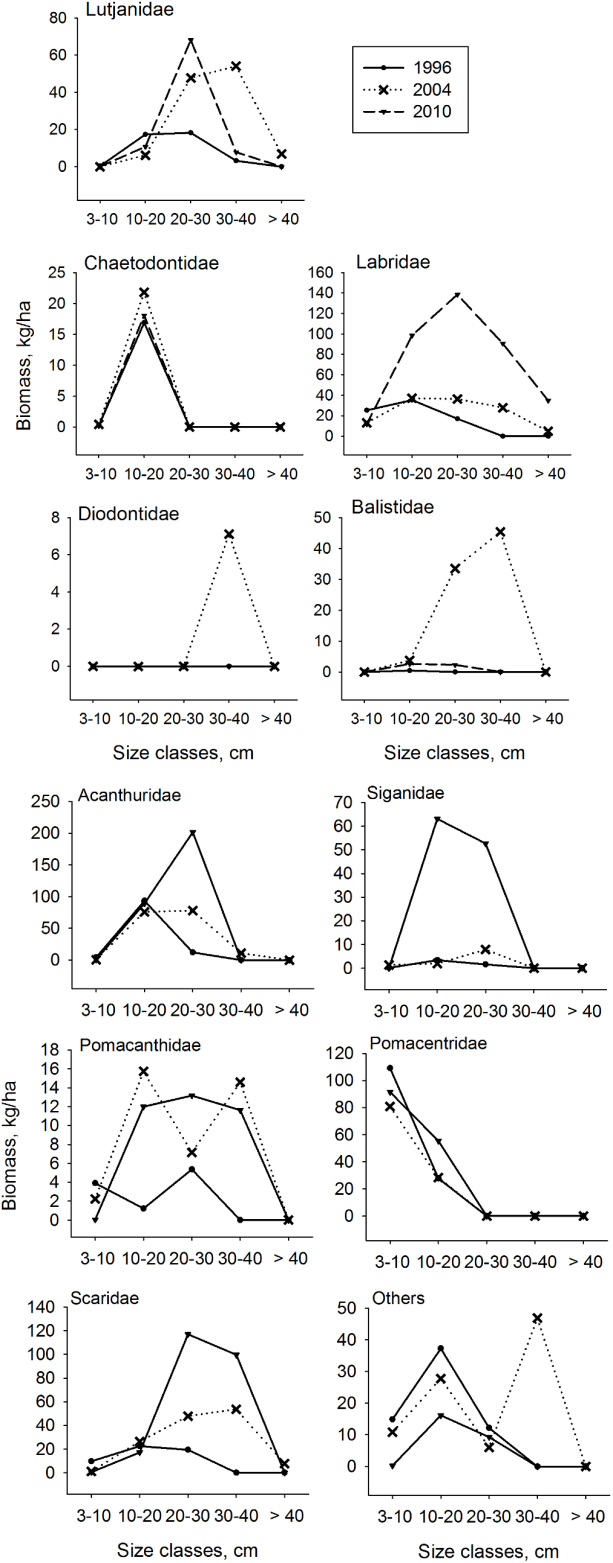
The changes in the biomass of finfish in different size classes. The biomass (mean kg/ha) was estimated for eleven common finfish families sampled in 1996, 2004 and 2010.

There was also variation in the biomass of different size classes between the sites. Over the three sampling periods, Funguni reef consistently had the highest biomass in all but the 3–10cm size class. Comparing only the results of the 2010 sampling period, Funguni reef also had the highest biomass in these size classes with the exception of the 3–10cm size class, followed by Maziwe and Dambwe reefs while Maziwe and Taa had the highest biomass of fish in the >40cm size class. The different fish families also showed variation in size with time in particular in the wrasses (3–10cm size class), in the butterflyfish, angelfish, and rabbitfish (10–20cm size class), in the triggerfish, snappers, and rabbitfish (20–30cm size class) and ballonfish (30–40 cm size class) (Fig 2).

#### Sea urchins

Eight species of sea urchins were recorded during the surveys and a high variation was observed in the number of species, their density and biomass between sites and with time (Table 5). All eight species were recorded at all reefs except *Echinothrix calamaris* that was only recorded at Funguni and *Tripneustes pileolus* that was only recorded at Taa reef.

**Table 5:** The biomass of sea urchin species (mean ± SD, kg/ha) sampled at Chanjale, Funguni, Makome and Taa in 1996, 2004 and 2010 and the results of Kruskal-Wallis tests of sites and of sampling periods (a), and the mean ± SD sea urchin predation index and the percent predation by fish and invertebrates in the sampled reefs and years.

a) Sea urchin biomass											
** **	** **	** Site**	** **	** **	** **	**Time **	** **	**Site**	** **	**Time**	** **
**Sea urchins**	**Chanjale**	**Funguni**	**Makome**	**Taa**	**1996**	**2004**	**2010**	**χ** ^**2**^	***P***	**χ** ^**2**^	***P***
*D*. *savignyi*	0	1106.5 ± 1413.0	129.6 ± 177.0	935.2 ± 696.2	1059.0 ± 1317.7	357.6 ± 547.9	169.4 ± 174.1	9.6	**0.022**	0.6	0.754
*D*. *setosum*	141.7 ± 283.3	11.1 ± 9.6	27.8 ± 48.1	77.8 ± 94.8	16.7 ± 23.5	45.8 ± 91.7	133.3 ± 244.7	0.9	0.819	0.95	0.622
*E*. *calamaris*	0	68.1 ± 78.7	0	0	12.5 ± 25.0	38.6 ± 77.1	0	7.2	0.065	1.4	0.503
*E*. *diadema*	578.3 ± 368.5	2788.9 ± 588.9	809.8 ± 598.5	925.3 ± 333.9	1378.4 ± 1416.1	1243.4 ± 986.2	1079.6 ± 822.0	7.5	0.057	0.1	0.958
*E*. *mathaei*	0	30.5 ± 25.0	0	8.6 ± 7.6	6.4 ± 7.6	17.4 ± 28.0	4.4 ± 9.8	9.4	**0.024**	0.7	0.690
*E*. *molaris*	0	9.8 ± 3.6	1.7 ± 2.4	12.7 ± 8.2	6.5 ± 8.0	8.1 ± 9.3	2.8 ± 2.8	9.5	**0.023**	0.8	0.675
*S*. *variolaris*	0	0	0	0	0	0	0	0	1	0	1
*T*. *gratilla*	0	100.9 ± 164.4	16.1 ± 14.0	12.1 ± 12.1	3.0 ± 6.1	84.8 ± 137.7	7.3 ± 4.8	4.2	0.245	3.6	0.165
*T*. *pileolus*	0	0	0	18.5 ± 23.1	11.1 ± 22.2	2.8 ± 5.6	0	7.2	0.065	1.4	0.503
**Total**	**720.0 ± 236.3**	**4115.8 ± 1885.7**	**984.9 ± 823.3**	**1990.2 ± 1125.7**	**2493.6 ± 2730.3**	**1798.5 ± 1404.2**	**1396.8 ± 867.4**				
b) Predation											
Predation index	0.23 ± 0.09	0.11 ± 0.06	0.19 ± 0.1	0.42 ± 0.09	0.29 ± 0.19	0.21 ± 0.14	0.2 ± 0.09	7.81	0.05	0.51	NS
Fish, %	57.5 ± 24.75	12.5 ± 17.68	60 ± 56.57	41.43 ± 2.02	-	45.71 ± 41.14	40 ± 24.83	2.91	NS	0.19	NS
Invertebrates, %	0	0	10 ± 14.14	17.14 ± 4.04	-	3.57 ± 7.14	10 ± 11.55	4.5	NS	0.83	NS

The overall numbers of sea urchins combined for sites that were sampled in all three sampling periods was significantly lower in terms of numbers (81%) and biomass (44%; Table 5) comparing 1996 to 2010. The large-bodied species *Echinothrix diadema* was the dominant sea urchin comprising 30% of the numbers and 65% of the biomass of all reefs combined followed by *Diadema savignyi* (35% and 28% respectively). There was high spatial variability in the sea urchins between reefs for the comparison of the four reefs that were sampled in the three sampling periods. Funguni reef had the highest density and biomass of sea urchins dominated by *E*. *diadema* while Chanjale reef had only two species of urchins *D*. *savignyi* and *E*. *diadema* that were recorded at low urchin numbers and biomass. There were no statistically significant changes in the biomass of individual species over time indicating species composition associations were possibly related with the specific reef condition. *Diadema savignyi* numbers did show a decline (-84%) but between-site variation was very high and other species showed little directional changes with time.

Of the reefs that were only sampled in 2010, Maziwe had fewer species but sea urchin numbers and densities were higher (3313 ± 1035.1 kg/ha, ±SD) than at Funguni reef (2693.1 ± 713.4, ±SD). Dambwe reef had sea urchin numbers and biomass (742.3 ± 231.2, ±SD) that were less than 50% the average for all reefs combined. In addition, comparing only the 2010 data, the biomass of urchins was higher (1975 ± 1289g/ha, ± SD) in the closures (Dambwe, Makome and Maziwe) than in the fished reefs (1416 ± 1109g/ha, ± SD).

Measurements of predation on *Echinometra mathaei* were completed at four reefs Chanjale, Funguni, Makome and Taa in all three sampling periods. Predation was low at all the reefs and there was no significant difference between reefs or between years. Fish were the dominant predators accounting for between 12 and 50% of the predation at all the sites.

#### Herbivory

Herbivory assays were conducted at Chanjale, Funguni, Makome and Taa in 2004 and in 2010 (Table 6). The rates of herbivory or % bitten varied weakly between the reefs and sea urchins were the dominant herbivore with 38% of the bites compared to 4% of the total for fish. In general, herbivory rates by types were proportional to the overall herbivory. Herbivory for all sites combined, exhibited a 38% non-significant decline between 2004 and 2010 reflecting large between-site variability. The amount of the assay eaten also varied weakly between reefs with low values in Makome and Chanjale and high values in Funguni and Taa (p = 0.04) (Table 6).

**Table 6:** Summary estimates of herbivory on the seagrass *Thallasia hemprichii* at Chanjale, Funguni, Makome and Taa in 2004 and 2010 presented as the mean (± SD) percent amount eaten, herbivory rate, the amount attributed to fish or to sea urchins and results of ANOVA tests of significance for differences between sites and time.

Category	Site				Time		Site		Time	
	Chanjale	Funguni	Makome	Taa	2004	2010	χ^2^	P	χ^2^	P
Herbivory, %	24.1 ± 15.2	47.0 ± 20.5	26.9 ± 19.1	73.33 ± 37.9	60.0 ± 28.7	37.4 ± 31.5	6.6	0.086	1.5	0.215
Amount eaten %	15.0 ± 13.1	36.9 ± 26.8	8.7 ± 8.7	59.7 ± 31.2	31.6 ± 30.3	29.1 ± 29.3	8.1	**0.044**	0.2	0.643
% urchin	21.9 ± 16.3	45.0 ± 20.6	22.4 ± 16.4	64.5 ± 38.4	45.7 ± 27.1	33.6 ± 30.4	6.4	0.095	1.3	0.261
% fish	2.2 ± 5.0	2.0 ± 4.5	4.4 ± 9.9	8.9 ± 8.8	5.3 ± 9.8	3.8 ± 5.7	2.7	0.440	0	1

#### Benthic cover

Benthic substrate composition varied among sites and hard coral, turf, and fleshy algal dominated comprising >82% of the overall substrate followed by coralline algae (8%), soft coral and sand (3–4%) (Table 7). There were also differences in the topographic complexity of the bottom with Funguni and Makome being more complex than Taa and Changale. Comparing all six reefs in 2010, Dambwe reef had the highest hard coral cover at 48.3%, while Funguni reef with the lowest cover (15.3%) cover of hard coral for all reefs combined. Among the sites sampled over time, hard and soft coral, turf, coralline, and fleshy algae, and sand varied strongly between sites (Table 7). There was variability in the benthic cover over time but, apart from an increase in topographic complexity, most other changes were not clearly in the direction of our working hypotheses. Amongst the algae, fleshy algae were as abundant as turf algae and varied weakly over time with the lowest values in 2004 and highest in 1996 and 2010. The calcifying red coralline algae generally showed an increase but most of the change was between 1996 and 2004 declining somewhat in 2010. Similarly, the calcareous green algae was not common but exhibited a weak directional increase over time.

**Table 7:** Benthic substrate composition (mean% cover ± SD) at Chanjale, Funguni, Makome and Taa reefs and the yearly averages for each substrate category for 1996, 2004 and 2010. A Kruskal-Wallis comparison of changes between sites and time.

Substrate	Site				Time			Site		Time	
	Chanjale	Funguni	Makome	Taa	1996	2004	2010	χ^2^	P	χ^2^	P
Hard coral, %	38.8 ± 12.6	13.5 ± 9	32.1 ± 22.4	18.8 ± 10.3	22.9 ± 20.1	27.8 ± 18.1	26.1 ± 14.1	38.6	< .0001	2.5	0.289
Algal Turf, %	24.5 ± 11.6	28.9 ± 13.4	32 ± 18.7	43.4 ± 9.5	35.8 ± 14.4	32.4 ± 18.1	28.5 ± 11.9	23.6	< .0001	4.6	0.10
Fleshy algae, %	18.5 ± 11	39.2 ± 14.8	26.6 ± 20.6	14.4 ± 10.1	27.2 ± 14.7	18.8 ± 14.9	28.8 ± 20.8	28	< .0001	6.8	0.033
Coralline algae, %	7.9 ± 7.1	11.3 ± 9.1	4.6 ± 5.3	7.6 ± 7.8	2.2 ± 5.1	13 ± 8.6	8 ± 4.4	8.7	0.033	41.3	< .0001
Soft coral, %	6.6 ± 6.2	2.4 ± 2.9	1.4 ± 3.4	5.1 ± 5.8	3.5 ± 4.1	3.7 ± 4.6	4.4 ± 6.6	24.5	< .0001	0.3	0.845
Sand, %	1.6 ± 3.5	1.5 ± 2.5	0.2 ± 0.8	7.4 ± 6.2	2.8 ± 5.3	2.1 ± 4	3.2 ± 4.6	37.4	< .0001	2.4	0.299
Sponge, %	0.6 ± 1.3	0.3 ± 0.6	0.3 ± 0.7	0.4 ± 0.8	0.3 ± 0.7	0.5 ± 0.8	0.4 ± 1.1	0.3	0.959	1.9	0.385
Calcareous algae, %	0.3 ± 0.6	0.4 ± 0.7	0.5 ± 2.2	0.03 ± 0.1	0.1 ± 0.2	0.3 ± 0.6	0.6 ± 2	7.7	0.052	5.1	0.077
Topographic complexity	1.23 ± 0.07	1.31 ± 0.09	1.17 ± 0.08	1.24 ± 0.09	1.21 ± 0.08	1.22 ± 0.09	1.28 ± 0.08	29.4	< .0001	13.2	0.001

The overall average hard coral cover for the closure sites (Dambwe, Makome and Maziwe) in 2010 was 37% and higher than non-closure reefs (21%). Makome reef, which was sampled in all three periods, exhibited a significant increase in 2004 from 28.6 ± 27.2% to 40 ± 22.1% hard coral cover, then a decrease to 27 ± 16.1% by 2010.

#### Coral community

A total of 31 coral genera were recorded in the surveys and while some genera including *Acropora*, *Galaxea*, *Porites*, *Millepora* and *Hydnophora* were common at all sites, the cover of individual genera varied with site and time (Table 8). The dominant genera at the reefs sampled in all three periods (pooled) were *Galaxea* that had an average cover of 22.5% ± 19.2 at Makome reef, *Montipora* with 13.4% ± 7.1, cover at Chanjale, massive *Porites* with 13% ± 2.9, cover at Taa and *Acropora* with 12% cover at Dambwe. *Galaxea*, *Montipora*, *Platygyra*, and *Stylophora* exhibited significant differences between-reefs while *Porites* branching and massive *Porites* exhibited weakly significant differences. The only significant difference between the genera with time was for *Synarea* that increased from 0.1 to 3.0% cover between 1996 and 2010 (Table 8).

**Table 8:** Hard coral genera at Chanjale, Funguni, Makome and Taa presented as the mean % (± SD) cover for each coral genera encountered along a 10m line transect in 1996, 2004 and 2010 and ANOVA comparison between sites and times.

Coral Genera	Site				Time			Site		Time	
	Chanjale	Funguni	Makome	Taa	1996	2004	2010	χ^2^	P	χ^2^	P
*Acropora*	2.8 ± 1.2	0.2 ± 0.2	0.3 ± 0.2	0.4 ± 0.04	0.7 ± 0.6	0.9 ± 1.4	1.2 ± 1.9	6.7	0.082	0.5	0.794
*Alveopora*	0	0.3 ± 0.4	0.02 ± 0.03	0	0	0.2 ± 0.4	0	1.3	0.734	7.2	0.028
*Acanthastrea*	0.3 ± 0.6	0	0.2 ± 0.4	0	0.2 ± 0.4	0	0.3 ± 0.5	2.2	0.53	1.1	0.573
*Astreopora*	0	0.1 ± 0.1	0.2 ± 0.4	0	0	0.04 ± 0.1	0.2 ± 0.3	2.2	0.53	1.1	0.573
*Coscinarea*	0.02 ± 0.04	0	0	0	0	0.02 ± 0.03	0	3	0.392	2	0.368
*Cyphastrea*	1.1 ± 1.5	0.03 ± 0.1	0	0.3 ± 0.4	0.02 ± 0.04	0.2 ± 0.2	0.9 ± 1.4	3.7	0.292	1.5	0.476
*Echinphora*	1.6 ± 0.9	0.4 ± 0.7	0.3 ± 0.5	0.3 ± 0.2	0.3 ± 0.4	1 ± 1.2	0.7 ± 0.6	5.9	0.118	1.5	0.471
*Favia*	0.7 ± 0.6	0.1 ± 0.1	0 ± 0	0.2 ± 0.3	0.4 ± 0.7	0.1 ± 0.2	0.3 ± 0.2	6	0.111	1.5	0.478
*Favites*	0.9 ± 0.6	0.3 ± 0.2	0 ± 0.1	0.3 ± 0.3	0.4 ± 0.4	0.2 ± 0.2	0.5 ± 0.7	6.3	0.098	0.6	0.759
*Fungia*	0.03 ± 0.1	0	0.1 ± 0.1	0.02 ± 0.04	0	0.1 ± 0.1	0 ± 0.1	3.7	0.293	1.1	0.584
*Galaxea*	2.7 ± 1.5	0.6 ± 0.5	22.5 ± 19.2	0.3 ± 0.03	7.3 ± 13.1	11.3 ± 18.6	1 ± 0.9	9.5	0.023	0.7	0.703
*Galaxea astreata*	0	0	3 ± 5.2	0	0	0	2.3 ± 4.5	3	0.392	2	0.368
*Gardineroseris*	0	0	0.1 ± 0.2	0	0	0	0.1 ± 0.2	3	0.392	2	0.368
*Goniastrea*	0.5 ± 0.6	0.1 ± 0.1	0.1 ± 0.2	0	0.1 ± 0.2	0.1 ± 0.2	0.3 ± 0.6	3.4	0.328	0.3	0.866
*Goniopora*	0	0.2 ± 0.1	0	0.1 ± 0.2	0.1 ± 0.2	0 ± 0	0.1 ± 0.1	3.9	0.269	2.4	0.303
*Hydnophora*	1.1 ± 0.9	0.7 ± 0.9	0.1 ± 0.1	0.2 ± 0.1	0.7 ± 0.9	0.5 ± 0.8	0.4 ± 0.5	4.7	0.198	1	0.594
*Leptastrea*	0.2 ± 0.2	0	0	0	0	0.05 ± 0.1	0.1 ± 0.2	6.5	0.088	1.1	0.573
*Leptoria*	0.5 ± 0.5	0.03 ± 0.05	0	0.2 ± 0.3	0.3 ± 0.5	0.02 ± 0.04	0.2 ± 0.3	4.2	0.243	1.3	0.524
*Lobophora*	0.3 ± 0.5	0	0	0.8 ± 1.4	0.2 ± 0.4	0	0.6 ± 1.3	2.2	0.53	1.1	0.573
*Millepora*	1.1 ± 1.2	1.6 ± 2.3	1.1 ± 1.7	1.3 ± 0.2	1.1 ± 1.1	1.4 ± 2	1.4 ± 1.2	0.8	0.852	0.1	0.944
*Montipora*	13.4 ± 7.1	0.8 ± 0.7	0.2 ± 0.4	0.1 ± 0.1	4.9 ± 8.6	4.5 ± 8.5	1.5 ± 2.5	9.1	0.028	0.2	0.901
*Pavona*	0.1 ± 0.1	0	0.9 ± 1.5	0.1 ± 0.1	0.1 ± 0.1	0.03 ± 0.1	0.7 ± 1.3	2.6	0.461	0.9	0.635
*Platygra*	1.7 ± 0.6	0.3 ± 0.2	0.1 ± 0.2	0.7 ± 0.3	0.8 ± 1	0.5 ± 0.4	0.8 ± 0.7	9.5	0.023	0.3	0.878
*Pocillopora*	0.6 ± 0.4	0.5 ± 0.3	0.3 ± 0.3	0.2 ± 0.3	0.5 ± 0.1	0.4 ± 0.5	0.3 ± 0.1	2.7	0.442	1.9	0.388
*Porites* branching	5.5 ± 0.9	1.1 ± 1.4	0.8 ± 1.1	0.3 ± 0.6	2.3 ± 2.5	1.7 ± 3	1.8 ± 2.1	7.2	0.067	0.6	0.732
*Porites* massive	3.1 ± 3.7	5 ± 3.7	0.5 ± 0.6	13.2 ± 2.9	4.4 ± 7.8	4.7 ± 4.8	7.2 ± 5	8.1	0.043	1.4	0.5
*Synarea*	0.3 ± 0.3	1.3 ± 2	1.7 ± 2.9	0.5 ± 0.6	0.1 ± 0.2	0.1 ± 0.1	2.6 ± 2.1	0.1	0.994	8.3	0.016
*Seriatopora*	0.2 ± 0.3	0	0	0	0	0	0.1 ± 0.2	3	0.392	2	0.368
*Stylophora*	0.4 ± 0.3	0.04 ± 0.1	0	0	0.2 ± 0.4	0.04 ± 0.1	0.1 ± 0.1	9.3	0.025	0.4	0.81
*Turbinaria*	0	0.02 ± 0.03	0.2 ± 0.3	0	0	0.01 ± 0.03	0.1 ± 0.2	2.2	0.53	1.1	0.573

### Social surveys

#### Respondent characteristics

Respondents were all males and there were significant differences between villages in terms of the mean ages and the years spent fishing, while there were no significant differences in the level of education, biweekly expenditure or the number of years the respondents lived in the community (Table 9). Kigombe village had the oldest and most experienced fishers, followed by Ushongo and lastly Mwarongo.

**Table 9:** Summary of key descriptors (mean ± SD) of the respondents in the three villages, results of a post-hoc Tukey test comparing individual village means, and the one-way ANOVA test of significance of the descriptors. There is no significant difference between the villages where values are preceded by the same letters in the post-hoc Tukey test.

	Sample size	Age of respondent	Level of education (yrs)	Biweekly expenditure, $	Years in occupation	Years lived in community
Villages						
Kigombe	24	*A* 46.0 ± 12.7	*A* 5.9 ± 2.8	*A* 62 ± 23.0	*A* 24.7 ± 13.5	*A* 40.5 ± 10.8
Ushongo	20	*AB* 43.2 ± 15.9	*A* 7.2 ± 2.9	*A* 57 ± 35.0	*AB* 19.7 ± 16.5	*A* 31.6 ± 20.4
Mwarongo	32	*B* 37.1 ± 2.2	*A* 7.0 ± 0.5	A 64 ± 5	*B* 16.0 ± 1.9	*A* 29.2 ± 3.4
Managers	3	42.3 ± 4.9	16.3 ± 1.5	216 ± 76.4	12.3 ± 12.7	14 ± 22.5
*ANOVA*						
*R* ^*2*^		0.07	0.03	0.009	0.07	0.06
*F ratio*		3.11	1.28	0.32	2.95	2.55
*P <*		0.051	NS	NS	0.053	NS

Exchange rate was 1470 TZS to 1USD in 2010

#### Perceptions of management options and benefits

**Fig 3:**
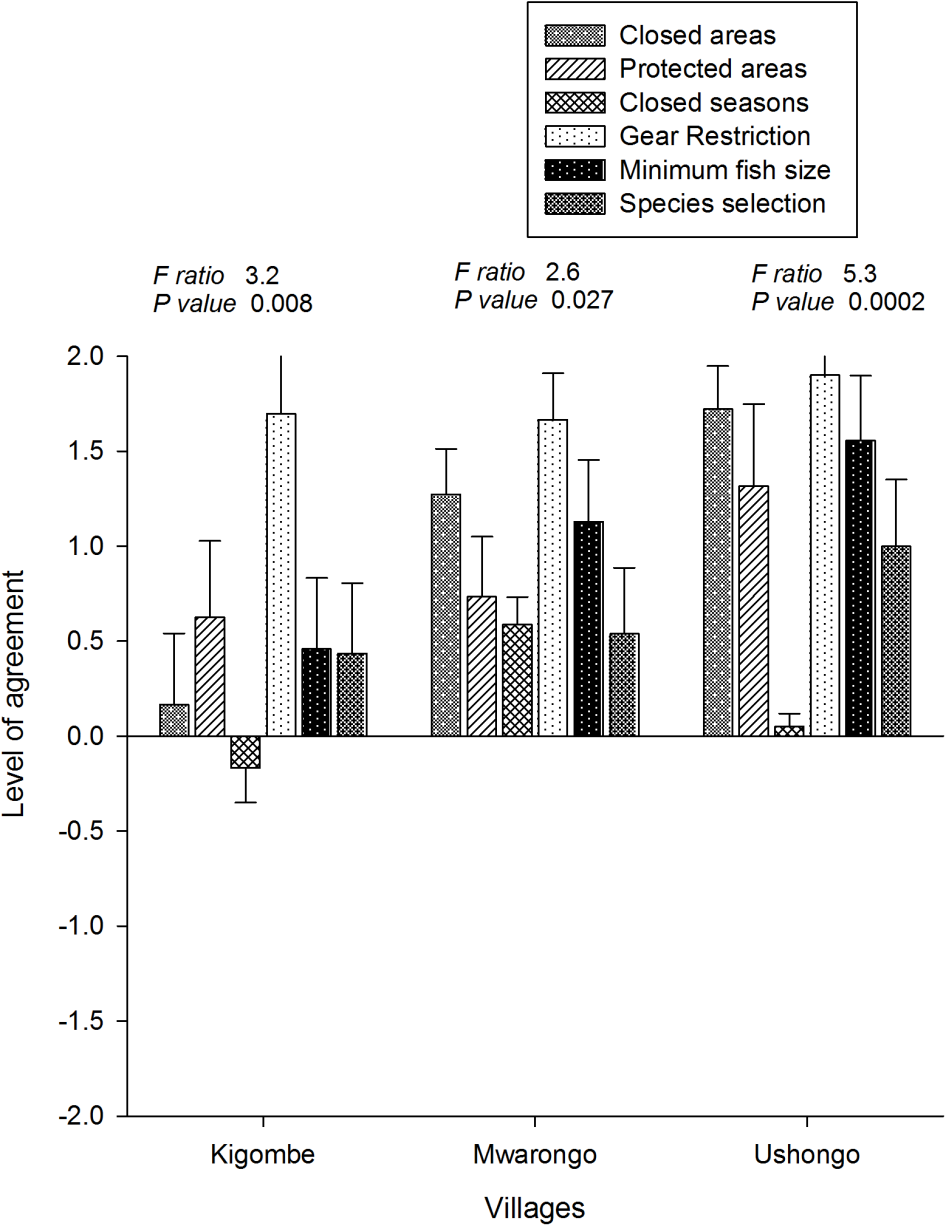
The level of agreement for six fisheries management options. The level of agreement (mean ± SEM; tests of significance) for six management options was calculated for respondents from Kigombe, Mwarongo and Ushongo villages.

There was high variability in the respondent’s and fishing villages perceptions of the ability of management options to improve fisheries (Fig 3; Table 10). Respondents from Ushongo village had the most positive perceptions and agreed that all restrictions improved fisheries with the exception of closed seasons. In contrast, respondents from Kigombe village did not perceive strong benefits except for gear restrictions, with closed areas and closed seasons receiving very low or negative scores. Mwarongo fishers were more intermediate and like other respondents gave positive responses to the gear restrictions. Overall, closed seasons and species selection were the least preferred options. Nevertheless, because of the high between-village variability, pooling them produced few statistically significant differences for any management options except closed areas, which had a low overall level of perceived benefits (χ² = 14.53, P = 0.024).

**Table 10:** Results of a forward stepwise regression analyses evaluating the association between socio-demographic and management restrictions variables. Results presented are (a) various management restrictions (b) communities, all fishers pooled.

a) Management restrictions				
**Management**	**Independent**	**F**	**P**	**Direction of**
**Restriction**	**Variables**			**association**
Closed areas	Perceived social disparity	25.15	< .0001	Negative
	Job diversity	2.18	0.103	Positive
Closed seasons	Job diversity	3.08	0.084	Positive
Gear restriction	Years in occupation	1.87	1.032	Positive
Minimum fish size	Perceived social disparity	1.23	0.265	Negative
Protected areas	Perceived social disparity	6.53	0.013	Negative
	Age of respondent	1.05	0.31	Negative
Species selection	Fortnight expenditure	7.57	0.007	Negative
b) Communities				
Kigombe	Perceived social disparity	31.72	**< .0001**	Negative
	Level of education	5.3	**0.023**	Negative
	Fortnight expenditure	8.52	**0.004**	Positive
Mwarongo	Years in occupation	7.33	**0.009**	Positive
	Level of education	7.88	**0.006**	Positive
	Fortnightly expenditure	2.03	0.157	Positive
	Material style of life	9.71	**0.002**	Positive
Ushongo	Years in occupation	1.87	0.174	Positive
	Job diversity	3.74	**0.056**	Positive

Respondents, who gave closed area management a high score, viewed these areas as fish breeding sites that could improve catches. On the other hand, respondents who were against closed areas revealed that the closed areas were a source of conflict as they reduced the size of the available fishing grounds. Respondents who had a low preference for closed seasons revealed that there were no alternatives to fishing in some seasons and respondents that gave species selection a low score revealed that this management intervention was not possible to implement given the type of gear in use.

**Fig 4:**
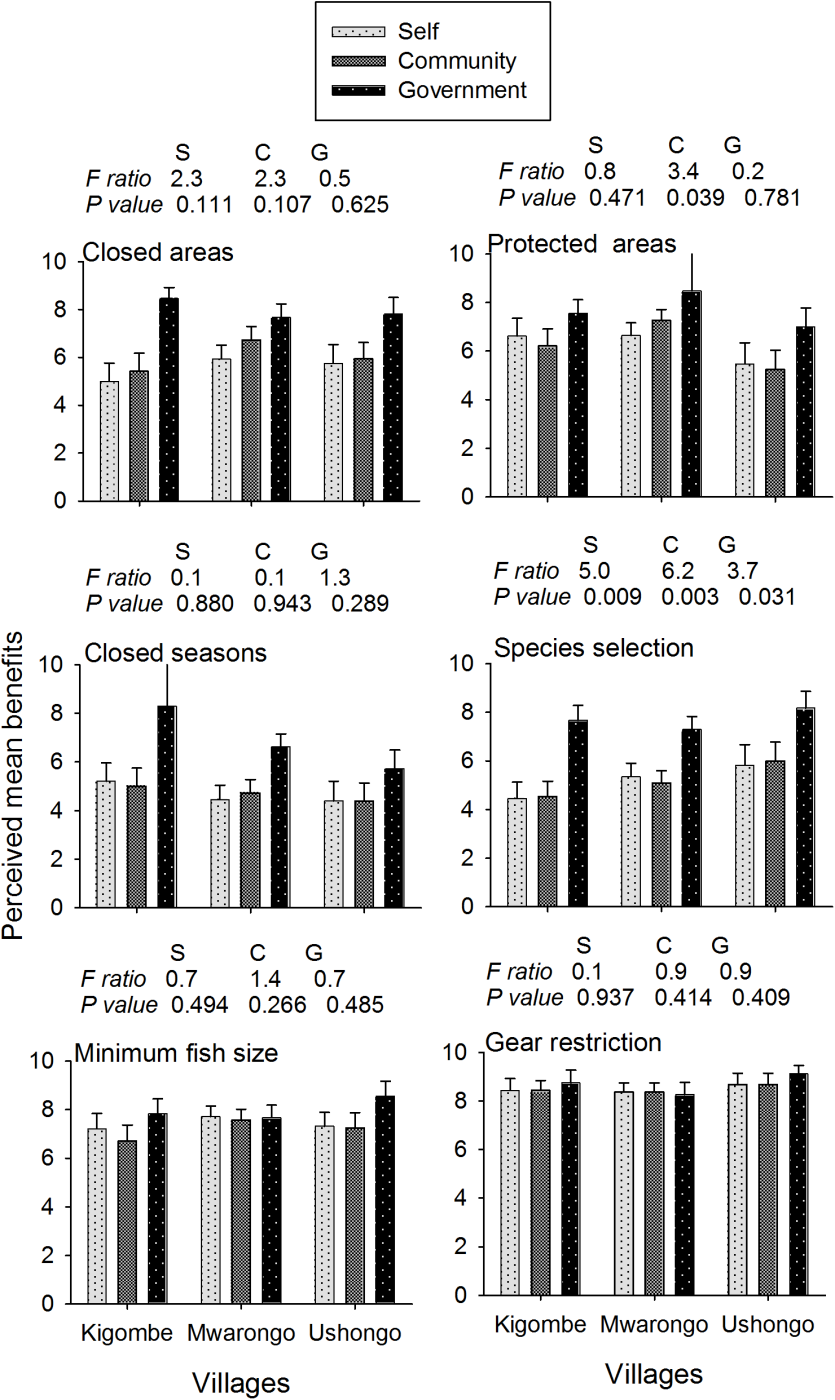
The perception of the level of benefits accruing to beneficiaries of fisheries management. The responses for perceived benefits (mean ± SEM; tests of significance) to the self, the community, and the government were estimated for six fisheries management options, for respondents from Kigombe, Mwarongo and Ushongo villages.

When the benefits of management were compared for the government, community and self, general management was viewed to be beneficial to all groups, on average, the government was perceived as the largest beneficiary of all the management options followed by the community and lastly the individual fisher (Fig 4). The gear restriction option was perceived to benefit all groups equally but disparity increased along a gradient from protected areas (1.3), closed areas (2.1), closed seasons (2.2), and species selection (2.5). Perceived mean disparity differed between villages and the village nearest the marine park headquarters, Kigombe, had the highest mean perceived disparity in all the management options except for the protected area option. In contrast, respondents from Ushongo village exhibited the highest disparities in the protected areas and in minimum fish size restriction options.

#### Perceived sustainability of management restrictions

Associations between individual socio-demographic variables with the scaled perceived sustainability of the various restrictions found that perceived social disparity, fortnight expenditure and age of respondent were negatively associated with perceived sustainability of restrictions while years in occupation and job diversity were positively associated with scaled perceived sustainability of restrictions (Table 10A). At the village level, we found that perceived social disparity was the strongest factor in Kigombe village followed by the fortnightly expenditure and level of education (Table 10B). Both the perceived social disparity and level of education were negatively associated with perceived sustainability of the management restriction, while the fortnightly expenditure showed a positive association. At Mwarongo village, the years in occupation, level of education, fortnight expenditure and material style of life were all positively associated with the perceived sustainability of various restrictions with material style of life and level of education having the strongest relationship. In Ushongo village, two factors, years in occupation and job diversity were both positively associated with management restrictions, while the response for years in occupation was not significant.

#### Proposed closure and minimum fish sizes

**Fig 5:**
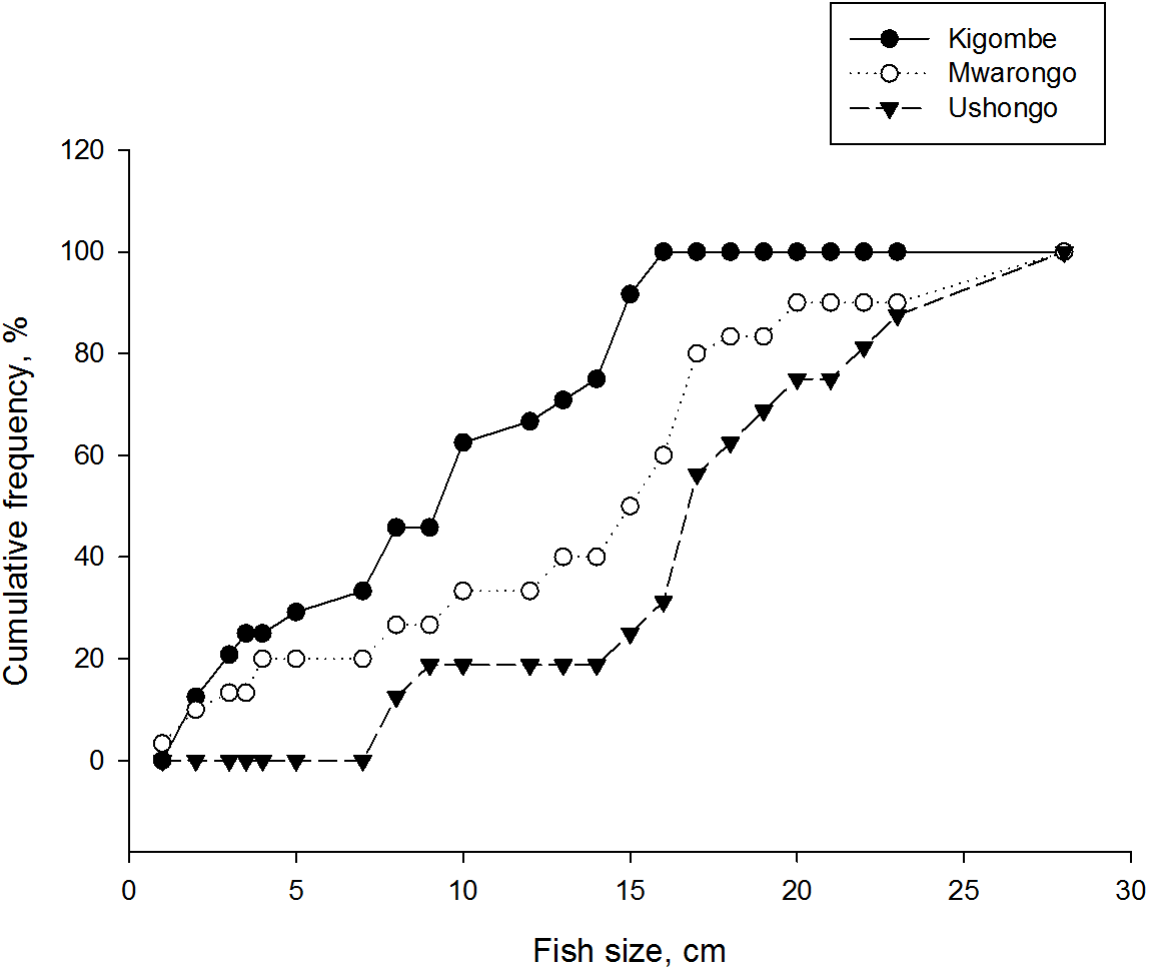
The preferred minimum fish size limit. The cumulative frequency of the preferred minimum fish size limit is plotted for respondents from Kigombe, Mwarongo and Ushongo villages.

When queried about details of minimum size limits, the villages differed significantly on the suggested minimum size of fish that should be caught with respondents from Ushongo, Mwarongo and Kigombe villages proposing 17.6 cm, 13.8 cm and 9.3cm minimum size limits, respectively (Fig 5, Table 11). Areas proposed for closures and protected areas also showed high variance (Table 11), and respondents from Kigombe proposed the larger protected area and smallest closures of the three villages. Mwarongo village respondents proposed the largest closure that was twice the suggested sizes at Kigombe and Ushongo villages.

**Table 11:** Summary of the means (± SD) and one-way ANOVA test of significance of respondent’s responses for the minimum sizes of fish (cm), the size of protected and closed areas (km^2^).

Villages	N	Minimum fish size	Protected areas	Closed areas
Kigombe	24	9.27 ± 5.0	5.16 ± 9.2	1.88 ± 3.9
Mwarongo	30	13.8 ± 7.5	2.37 ± 5.6	8.56 ± 31.3
Ushongo	16	17.6 ± 6.0	1.69 ± 4.1	2.16 ± 3.5
*ANOVA*				
*F ratio*		*8*.*52*	*0*.*67*	*0*.*51*
*P value*	* *	***0*.*0005***	*0*.*522*	*0*.*602*

## Discussion

### Changes in reef ecology

The results indicate moderate variability but some ecological changes that are consistent with an improved ecology expected to occur with the compliance of increased management restrictions. Specifically, there was an increase in the larger body sizes of a number of targeted fish groups, an increase in overall fish biomass, a downward trend in total sea urchin numbers and biomass, and a decline in turf and increase in calcifying algae [17]. Coral cover remained stable but given the many declines in coral cover reported for the region over this period, this indicates an overall positive finding [29, 30]. The study was undertaken before and after the 1998 temperature anomaly, which was one of the deadliest disturbances for corals in recent history [31]. Kenyan reefs just to the north experienced a 45 to 70% decline in coral cover over this period and many changes in the taxonomic composition [32, 33]. Consequently, there is cumulative evidence that the ecological state of these reefs improved across and after the activities of this donor project and that there may be some factors conferring resilience to disturbance among these reefs [34].

Improved fisheries management is expected to allow targeted fish to grow to larger sizes and this is expected to change the taxonomic composition of the fish community towards slower growing species with larger body sizes and longer lives [35, 36]. The increased reproductive output due to larger body sizes with the potential for fisheries recovery has been hypothesized to be important for the recovery of overexploited fisheries [33]. Increased size may result in some declines in the smaller size classes and species susceptible to greater competition or predation [37]. We did observe the largest increases of fish in the 10–20 cm size category, which probably represents the size class that are frequently targeted by fishing and are sufficiently abundant to detect changes [38]. The larger classes did indicate increases over time but numbers were fewer, variability high, and this can result in weak detectability of fishing and recovery impacts. Nevertheless, rabbitfish, one of the most prized species, showed the largest gain in numbers consistent with their high fishing mortality and growth rates [39]. Wrasses and parrotfish also exhibited fast recovery, with their biomass increasing by ~ 5 times in 14 years. This is consistent with recovery studies in the region that indicate moderate to high growth of these taxa after the cessation of fishing [40].

Triggerfish and surgeonfish have been shown to recover more slowly and the surgeonfish response here was consistent with past reports, their biomass increased ~2 times in 14 years. The triggerfish family was probably most influenced by an episodic recruitment pulse of one species, *Sufflamen fraenatus*, in the middle of the study followed by a subsequent decline. Consequently, this indicates some species- and environment-dependent responses that make it difficult to generalize at the family level. *Sufflamen fraenatus* is a micro- rather than macro-invertebrate predator, and not an important sea urchin predator or expected to cause a decline in sea urchin abundance [41]. Nevertheless, wrasses, such as species in the genus *Cheilinus* and *Coris* that feed on sea urchins and rapidly recover, can play a role in reducing sea urchin numbers. It should also be appreciated that species-specific responses to declining fishing pressure are expected for parrotfish. Parrotfish have been shown to exhibit different responses to fishing and recovery rates with scraping species responding more than grazing and detritivorous species [36, 42, 43]. This is likely to have implications for the rates and types of ecological changes that can be expected over a 14-year period.

Improved management was also expected to lead to an increase in predation and herbivory rates, a decline in sea urchin biomass, and an increase in calcifying algae and decline in non-calcifying algae. There was some evidence for a cascading ecological effect that included the changes in the sea urchins, herbivory and benthic community but these were not strong and the evidence equivocal. Specifically, there was a ~40% decline in sea urchins numbers and a decline in herbivory by sea urchins and evidence for an increase in calcifying algae [17, 44]. Yet, erect fleshy algae were generally high in all reefs, did not steadily decline, and reached their highest levels in 2010. There was no evidence that herbivory by fish increased over the 2004 and 2010 period, which may result from an insensitivity of the herbivory assay or be a slower response of herbivorous fish to reduced fishing intensity, as found in some Kenyan fisheries closures [44, 45].

If predation on sea urchins were to increase with increasing fish biomass, the prediction is that the intermediate sized *Diadema* would decline before the large-bodied *Echinothrix diadema*. There was no evidence of increased predation, weak evidence that the abundant *D*. *savignyi* declined but no evidence for the uncommon *D*. *setosum* and high between-site variability precluded any conclusions. Additionally, the intermediate-sized and seagrass associated *T*. *gratilla* displayed a strong increase in the middle of the study period, suggesting a species or environment-driven pulse in numbers. Finally, total coral cover was mostly unchanged except for compositional changes and the increase in *Synarea*, which is a taxa tolerant of warm temperature disturbances [32]. While pooled coral cover was higher in the closures in 2010, this could be due to selecting sites with high coral cover for protective management rather than management impacts. Overall, the moderate increase in fish abundance and the time scale of this study may have been insufficient to measure large ecological changes observed in this region with increasing fish biomass [46].

Overall fish biomass increased in the studied reefs by ~400 kg/ha in 15 years, suggesting an ~10% net increase per year. This increase comes from both endogenous growth and net immigration into the reef ecosystem. McClanahan et al. [40] estimated a maximum fish production of 140–160 kg/ha/y for Kenyan reefs when fish biomass is reduced to low levels. If these values are transferable to the nearby Tanga fishery, we can estimate that Tanga fishers captured two thirds of the net production while one third remained and contributed to the recovery of fish biomass. Consequently, the Tanga fishery was probably producing high catch rate of 100 kg/ha/y (10 tons/km^2^/y) while still contributing to ecological recovery. The implication is that if the widespread use of seine nets and dynamite is curtailed, damaged reefs can still produce food and recover. This is hopeful for resolving the destructive fishing-poverty-conservation dilemma [47]. It indicates that maintaining high levels of fish production and improving fish and reef communities is possible in poor tropical countries when there is agreement and compliance with gear restrictions over extended periods of time.

### Perceptions of management restrictions

Resource users were generally supportive of restrictions and acknowledged benefits but their support of specific restrictions was highly variable and particularly between the studied fishing villages. Most agreed to the benefits of gear and size restrictions but there was considerable disagreement over the value of protected areas, closed areas, seasons, and species restrictions. The perceived benefits of restriction were generally aligned with the stakeholder scale of who benefits, with some of the more contentious benefits perceived to largely accrue to the government. This was particularly true for Kigombe village, which was close to the marine park headquarters and may reflect a tension or conflict between managers and resource users over resources accrued from specific management interventions. Kigombe fishers were also on average older, had more years fishing, and were the least educated of the three villages, which are socioeconomic factors often associated with negative perceptions towards some strong restrictions [27]. These types of between-village differences were also reported for Mafia Island, Tanzania and in some locations near protected areas in the region [26, 48, 49]. This indicates that nearness of fishers to protected areas with closures may accentuate the benefits and perceived inequality that might arise from protected area resource management. It also suggests the potential for conflict over resource restrictions when adjacent villages are not in strong agreement. Dynamite fishing in this region was frequently blamed on other villages, for example [50].

## Conclusions

The small fisheries closures appeared to have little effect on fish biomass but there was a clearer increase in fish sizes that indicates a positive effect of gear management, specifically the major reduction in beach seine numbers and dynamite use. Consequently, reducing these two drivers was likely to have had the most impact in improving the condition of reef fishes. This finding also appears to align well with fishers’ perceptions of the benefits of these restrictions and is likely to reflect either their empirical observations or willingness to comply for potentially self-interested reasons. Additionally, the lack of change in the hard coral community suggests resilience and some unknown, weak or interactive effects of sea urchins, algae, destructive fishing methods, and the 1998 temperature anomaly. Despite the concern about dynamite and dragnets in the region [51], we did not record any changes in coral cover or species losses in our 14-year study. Dynamite fishing is focused on fish aggregations that are found on coral reefs and is expected to damage corals but the frequency relative to the recovery rate will be critical to detecting impacts [52]. A single blast can be indistinguishable from background sites after five years but continuous blasting can result in rubble fields [52]. Consequently, perhaps the frequency of this disturbance in these reefs is lower than the recovery potential but given the lack of empirical studies, the impacts remain speculative. The lack of significant change in coral cover contrasts with the losses reported more broadly in the Indian Ocean region where temperature anomalies are the most common cause of coral loss [30]. The study does not allow for definitive understanding of social-ecological causation but increased compliance with gear restrictions is likely to have contributed to the improvements in reef ecology. Gear restrictions find support in the interviewed communities, which is expected to result in high compliance with the potential for improved ecological condition. Consequently, gear restriction is likely to be the form of management that will persist into the future if promoted and enforced, which is expected to assist the persistence of these reefs’ ecological functions.

While donors and their actors were involved in many activities in this location ranging from many community, government, and judiciary meetings and various levels of training and implementation [14, 18, 50], it is likely that many activities did not have considerable residence. For example, despite many community meetings and trainings we found heterogeneous disagreement about the benefits of many management options at the time of our interviews or three years after the project closed. Additionally, some limited use of seine nets and dynamite have been reported in the area after the project ended [51]. There are many possible explanations for this reversal of management including disbelieve, cynicism, empirical experience, poverty, short-term outlooks, poor enforcement, corruption, perceived inequity, and lack of benefit sharing. Additionally, despite the reported success on the scale of our study, there is no assurance that the improved management and social-ecological conditions will persist. Nevertheless, this study does provide one of the more quantitative and better-designed evaluations of marine conservation and management donor project in Africa that needs to be replicated more broadly and over larger scales of time and space [8]. These findings also suggest the need to continue adaptive monitoring and enforcing agreed upon regulations and resource status beyond or with continued small post-development donor support. While the establishment of the management system is considered typical of development that requires foreign assistance, persistent lower level involvement is likely to be needed to insure that the positive outcomes of the development project persist beyond the main funding cycle.

## Supporting Information

S1 Dataset Dataset A contains the dataset of the biomass (kg/ha) of common finfish families at the studied reefs. Dataset B contains the dataset of the biomass (kg/ha) of finfish in different size classes at the studied reefs. Dataset C contains the dataset of the biomass (kg/ha) of sea urchin species at the studied reefs. Dataset D contains dataset of the cover (%) of coral genera encountered along a 10m line transect at the studied reefs. Dataset E contains the dataset of the benthic substrate composition (% cover) along a 10m line transect at the studied reefs. Dataset F contains the dataset of the estimates of herbivory on the seagrass *Thallasia hemprichii* at the studied reefs. Dataset G contains dataset of the key socioeconomic descriptors of respondents at the villages sampled. Dataset H contains a dataset for evaluating the association between socio-demographic and management restrictions variables. Dataset I contains a dataset of respondent’s responses for the minimum fish size, protected area size, and closed area size at the villages sampled. (XLSX)
